# Users' perspectives of barriers and facilitators to implementing EHR in Canada: A study protocol

**DOI:** 10.1186/1748-5908-4-20

**Published:** 2009-04-09

**Authors:** Marie-Pierre Gagnon, Nicola Shaw, Claude Sicotte, Luc Mathieu, Yvan Leduc, Julie Duplantie, James Maclean, France Légaré

**Affiliations:** 1 Research Center of the Centre Hospitalier Universitaire de Québec, Québec, Canada; 2 Department of Nursing, Université Laval, Québec, Canada; 3 Faculty of Medicine, University of Alberta, Alberta, Canada; 4 Department of Health Management, Université de Montréal, Montréal, Canada; 5 Department of Nursing, Université de Sherbrooke, Québec, Canada; 6 Department of Family Medicine, Université Laval, Québec, Canada; 7 Innovation and Adoption Committee, Canada Health Infoway, Canada

## Abstract

**Background:**

In Canada, federal, provincial, and territorial governments are developing an ambitious project to implement an interoperable electronic health record (EHR). Benefits for patients, healthcare professionals, organizations, and the public in general are expected. However, adoption of an interoperable EHR remains an important issue because many previous EHR projects have failed due to the lack of integration into practices and organizations. Furthermore, perceptions of the EHR vary between end-user groups, adding to the complexity of implementing this technology. Our aim is to produce a comprehensive synthesis of actual knowledge on the barriers and facilitators influencing the adoption of an interoperable EHR among its various users and beneficiaries.

**Methods:**

First, we will conduct a comprehensive review of the scientific literature and other published documentation on the barriers and facilitators to the implementation of the EHR. Standardized literature search and data extraction methods will be used. Studies' quality and relevance to inform decisions on EHR implementation will be assessed. For each group of EHR users identified, barriers and facilitators will be categorized and compiled using narrative synthesis and meta-analytical techniques. The principal factors identified for each group of EHR users will then be validated for its applicability to various Canadian contexts through a two-round Delphi study, involving representatives from each end-user groups. Continuous exchanges with decision makers and periodic knowledge transfer activities are planned to facilitate the dissemination and utilization of research results in policies regarding the implementation of EHR in the Canadian healthcare system.

**Discussion:**

Given the imminence of an interoperable EHR in Canada, knowledge and evidence are urgently needed to prepare this major shift in our healthcare system and to oversee the factors that could affect its adoption and integration by all its potential users. This synthesis will be the first to systematically summarize the barriers and facilitators to EHR adoption perceived by different groups and to consider the local contexts in order to ensure the applicability of this knowledge to the particular realities of various Canadian jurisdictions. This comprehensive and rigorous strategy could be replicated in other settings.

## Background

Although the electronic health record (EHR) is a clear priority for policy makers in Canada [1], this country is still lagging behind other industrialized countries in that respect [2]. Federal, provincial, and territorial governments, in partnership with Canada Health Infoway ('Infoway'), are currently developing an ambitious project consisting in the implementation of a pan-Canadian interoperable EHR, that is, an EHR that has the ability to work with other information systems across organizations [3]. Infoway's program focuses on implementing a network of interoperable EHR solutions in Canada linking medical clinics, hospitals, pharmacies, and other points of care. This integrated patient-centred health record provides a longitudinal view of an individual's key health history and care, including physician visits, hospitalizations, diagnostic images and reports, laboratory test results, prescribed drugs, and immunizations. It will give authorized healthcare providers rapid access to patients' complete, up-to-date health information to support clinical decision-making and case management.

The EHR is considered as the key to the integration of various tools (*e.g*., test ordering, electronic prescription, decision-support systems, digital imagery, telemedicine) that will enable a safer and more efficient healthcare system for every Canadian [4-6]. Benefits for patients, healthcare professionals, organizations and the public in general are expected. It is argued that the EHR has the potential to fill the information gaps that are believed to currently compromise the quality and productivity of Canada's healthcare system [7]. One of the main benefits reported is the increased quality of care resulting from patients having their essential health data accessible to their different providers [8,9]. Also, the EHR could support empowered citizens to actively take part in decisions regarding their health, based on relevant disease management programs [10]. However, as Richard Alvarez, Infoway's President and CEO, asserts: 'we can only succeed in making electronic health records a reality if health care providers adopt the technology. Without their acceptance, our efforts are futile' [11].

### Challenges to implement EHR in a complex healthcare system

Many previous EHR projects have failed due to the lack of integration into practices and organizations, thus making the implementation of EHR a timely and foremost important issue [12,13]. A comparative study of EHR adoption among general practitioners (GPs) in 10 countries showed that Canadian GPs ranked last [2]. This huge lag in implementation of EHR points to the need of identifying its contributing determinants from macro-level systemic factors to micro-level individual barriers.

Perceptions of EHRs may vary between health professionals groups, adding to the complexity of implementing this technology in a pluralist healthcare system. Furthermore, discrepancies between patients' and professionals' perspectives may obstruct the adoption and use of EHR [9]. Given the lack of current evidence on effective strategies to implement interoperable EHR, there is an urgent need to synthesise knowledge regarding the integration of this complex and innovative technology into current practices. Therefore, it is imperative to conduct a knowledge synthesis of the factors that could affect the implementation of an interoperable EHR in the healthcare system given the multiplicity of providers involved. Furthermore, perspectives of patients and the public in general regarding an interoperable EHR have rarely been addressed and deserve specific attention.

### EHR and patients and public participation

As the role of patients in making decisions regarding their health and influencing healthcare policies increases, their expectations and demands will be a major force in driving the use of EHR [14-16]. While patient safety is 'a cornerstone of Infoway's activities' [5], patient and public participation in decisions regarding EHR implementation has been limited. Nevertheless, literature supports the benefits of patients' involvement in the development of EHR. For instance, patients accessing their primary care EHR in the UK had an increased perception of security and privacy [17]. Patients also expressed their interest in having more features available on their EHR, but were concerned about the nature of the information accessible to different healthcare providers. The EHR aims to collect information to allow for 'cradle to the grave' treatment [18]; thus, health consumers are major players in ensuring that this will come to fruition [19]. A survey has shown that the Canadian population was ready for a greater use of healthcare Information and Communication Technologies (ICT), but that citizens had several doubts and concerns that should be taken into account in order to achieve the full potential of these technologies [20].

### Gaps in knowledge that this study is addressing

Up to now, studies on the factors that affect EHR implementation have mostly focused on healthcare professionals, especially physicians [21]. A majority of these studies presented methodological limitations and findings have been dissonant [22]. Comparisons of the perspectives of various professional groups (nurses, pharmacists, medical archivists, managers, *et al*.) toward the EHR have been reported in the literature, but this knowledge has not been synthesized [23]. In a healthcare system that tends toward greater interdisciplinarity [24], it is critical to acknowledge the dynamics of each groups of users and their interdependence when introducing the EHR.

### Goal and objectives

We propose a method for developing a comprehensive and inclusive synthesis of actual knowledge on barriers and facilitators influencing EHR implementation in order to conciliate opinions from diverse groups of stakeholders, including patients and the public. This knowledge will help decision makers elaborate effective strategies to support the implementation of EHR in Canada by informing them on the key issues that should be taken into account.

The chief objective of the proposed study is to develop a novel method for producing a comprehensive and inclusive knowledge synthesis that will ultimately facilitate evidence- and context-based decisions regarding the implementation of EHR in the healthcare system. The specific objectives are to: conduct a mixed-method review of the literature on the barriers and facilitators related to the adoption of an interoperable EHR among the targeted groups of users (public, patients, healthcare professionals, managers); categorize, synthesize, and compare the perceptions of these different groups; underline the adoption/non-adoption factors specific to each professional group (physicians, nurses, pharmacists) and those specific to collective and interdisciplinary clinical work; and identify key issues for interoperable EHR implementation relevant to the specific context of the Canadian healthcare system. This knowledge synthesis will lead to a proposed set of strategies for effective implementation of EHR in Canada by identifying its barriers and facilitators.

## Methods

The guiding principle of this knowledge synthesis is its applicability to answer real challenges that decision makers face in implementing EHR. The project is divided into two main phases that will allow: reviewing and synthesizing relevant literature on barriers and facilitators to implementing the EHR that are perceived by its various end-users and beneficiaries; and validating these findings for the implementation of EHR in Canada.

### Systematic review of barriers and facilitators to EHR adoption

During the first phase of the study, a comprehensive review of the scientific literature (qualitative, quantitative, and mixed-methods studies) and other published documentation (technical or 'grey' literature) on the barriers and facilitators to the implementation of the EHR will be conducted. Previous reviews and syntheses conducted in the field of healthcare ICTs [25-30] have guided the elaboration of the search strategies.

### Sources of data

Standardized literature searches will be conducted on all relevant databases (MEDLINE, Ovid, Cochrane Central Register for Controlled Trials, Campbell Collaboration Register for Controlled Trials, Current Content, Science Citation Index, Social Sciences Citation Index, LISA, CINAHL, PsychINFO, EMBASE, Electronics and Communications Abstracts, Computer and Information Systems Abstracts, ERIC, ProQuest). The sensibility of the search strategy will be validated by ensuring that all relevant key articles identified by all team members (including decision makers and researchers) are retrieved. Relevant references from studies found through the above routes will be followed up and obtained for assessment. Other literature will be identified through internet search engines and government websites. Hand searches will be performed in specialized scientific journals with a focus on healthcare ICT and implementation research. Finally, publications citing the selected articles as well as other articles from authors of the selected articles will be searched through the ISI Science Citation Index. Specialized email lists will be used to contact experts in the field of EHR for unpublished studies. The diversity of interests and expertise among researchers of the team and their respective networks will ensure that all relevant literature is covered.

### Inclusion/exclusion criteria

#### Type of studies

All rigorous quantitative, qualitative, and mixed-methods designs will be considered. We will use specific scales to assess the quality of each type of design, based on a recent tool that proposes specific criteria for assessing quantitative (experimental and observational), qualitative, and mixed-method designs [31]. The following Studies published in all languages will be included.

#### Participants

Professional groups included are: physicians, nurses, pharmacists, medical archivists, and managers given that they are potentially the greatest users of the EHR in the Canadian healthcare system [32]. Studies aimed at EHR implementation from the perspectives of patients, health consumers or the general public will also be included.

#### Intervention

The implementation of any interoperable EHR will be the targeted intervention. We will consider 'interoperable' EHR as long as there is an exchange of health data involving more that one organization and/or setting of care.

#### Objective

Included studies must clearly mention a focus on barriers and/or facilitators to EHR implementation. A structured data collection process must be clearly described, *i.e*., research strategies and measurement tools in relation to the study methodology must be present. Thus, studies reporting unstructured observations, editorials, comments, or position papers will be excluded. Systematic reviews and meta-analyses will be considered if their main focus is on barriers and facilitators to the adoption of EHR.

#### Screening and data abstraction

All titles and abstracts will be screened independently by a team consisting of one of the two principal investigators (MPG and FL) and a research associate to assess which studies fit the inclusion criteria. Any discrepancies between the two reviewers on study inclusion will be resolved by discussion with other team members. Full text copies of all potentially relevant papers will be retrieved. Then, each study will be independently abstracted by two reviewers. For each group of EHR users identified, barriers and facilitators will be categorized and compiled using a validated extraction grid. This grid has been developed by one of the investigator (FL) [33,34] and combines various factors that are likely to affect healthcare professionals' behaviours identified from existing conceptual frameworks [35-37]. The grid has been validated for a study of barriers and facilitators to the implementation of shared decision-making in clinical settings [26]. Specific adaptations will be made to the grid in order to ensure its applicability to studies reporting the perspectives of patients and citizens toward the EHR. The grid will help organizing the information by providing a preliminary classification of the various barriers and facilitators to the adoption of EHR.

#### Appraisal of study quality

The quality of all eligible studies will be assessed by the two independent reviewers using quality criteria specific to quantitative, qualitative, and mixed-methods designs [38-40]. Studies that do not meet a minimal quality threshold on their respective quality scales will be discarded. Any discrepancies in quality ratings will be resolved by discussion and involvement of an arbitrator among other team members when necessary. Technical and grey literature will also be appraised for quality, but given that there are no consensus criteria for quality for this literature that we are aware of, studies from these sources will be considered as complementary to the scientific literature.

#### Study relevance screening

To ensure their relevance to inform decisions on EHR implementation, all eligible studies will be assessed by two independent reviewers representing decision makers. The four dimensions of user-based relevance proposed by Saracevic [41] will be used: aboutness (referring to how well the topic of the study matches the objective of the review), pertinence, context, and motivation. However, to the best of our knowledge, there are no specific scales or checklists available to measure those dimensions. We will identify criteria through a deliberation process with twelve to fifteen decision makers (physicians, nurses, medical archivists, managers and patients, from eastern, western, and central provinces, as well as territories of Canada). This unique process will favour interactions between researchers and decision makers on studies' relevance regarding their use to support real life decisions. When a consensus on the criteria for each dimension will be obtained, a scale will be created and face validated with members of the innovation and adoption committee of Infoway (where decision makers from all Canadian provinces and territories are represented). This scale will be used independently by two reviewers among the decision makers collaborating to the research team to assess the relevance of each study. Any discrepancies in relevance ratings will be resolved by discussion and involvement of another reviewer where necessary.

#### Methods for synthesising findings

Findings will be reported using consensual guidelines for narrative syntheses and meta-analytical techniques [42-45]. Factors identified will be grouped according to the underlying theoretical concepts. An iterative analytical method will be performed, based on transparency and search for consensus between the reviewers. Thus, other emergent categories of barriers and facilitators might be added to the classification grid during the review process. A narrative synthesis [42,44] will be performed to summarize the evidence from various types of studies. A comparison of the barriers and facilitators to EHR adoption among the various groups represented will be done using meta-analytical techniques. Results will be presented according to each professional groups and health consumers for which barriers and facilitators to the EHR adoption have been studied. Also, factors that are specific to interdisciplinary clinical work will be clearly identified. This synthesis will provide insight on a wide range of conditions that might influence the acceptance, adoption, utilization, and integration of an interoperable EHR in the healthcare system.

#### Strategies to ensure methodological rigor

Guidelines from recognized organizations, such as the Cochrane and the Campbell Collaborations, will be followed for ensuring the methodological rigor of this systematic review. Given the variability in the nature of the literature that will be assessed through this review, we will make sure that appropriate criteria are used to assess the quality of each type of studies (quantitative, qualitative, and mixed-methods). Both principal investigators have experience in mixed-methods systematic reviews of healthcare professionals' behaviours [25,26,34,46,47] that will help in structuring the synthesis process. Furthermore, the research team combines different types of expertise that will enable a comprehensive synthesis of the knowledge on factors affecting the adoption of the EHR from various perspectives.

#### Pan-Canadian Delphi study

To help ensure their contextualization and relevance for policymaking in the Canadian context [48], findings from the systematic review will be presented to a large panel of experts in the second phase of the study. This represents a novel approach to knowledge synthesis because it aims to increase the relevance and applicability of scientific evidence for decision makers [48]. A Delphi study [49] involving representatives from each group will be conducted with the aim of reaching a consensus between those experts. This type of study is highly recommended for obtaining opinions from experts who live and work in different geographic regions and settings [50]. The anonymity of the Delphi process also encourages open and honest feedback among experts.

#### Selection and recruitment of the expert panel

The aim of the Delphi study is to obtain opinions from each group of EHR users representing a variety of expertises and contexts in Canada. As such, 10 to 18 experts [49] for each group of EHR users will be identified across Canada through professional associations and corporations, regional health authorities, and regional EHR team projects from each province. A list of potential participants will be created through the contact networks method [51], with the help of decision makers of the team and their collaborators. Recruitment of experts will be done by email. A postcard will also be sent to allow contacting participants who do not have email or do not use it, to limit possible selection bias. However, participants must have internet access to be included in the study. The message will present the study's objectives, the nature of their implication, and will solicit their participation in the Delphi study. The message will also provide a link to the study website (or URL address in the postcard) and give participants a temporary username.

#### The Delphi process

The first step of this Delphi study is to develop a pre-test questionnaire from the findings of the systematic review. This questionnaire will present the principle barriers and facilitators that have been reported in the literature for each group of EHR users. The selection of items will be based on their relative importance in the literature. In general, items mentioned by 15% or more of the studies will be kept, based on content analysis techniques to identify salient beliefs in the construction of questionnaires [52]. Experts representing groups of EHR users will validate this questionnaire to ensure the good understanding of the questions and to evaluate the time needed to complete it. After this pre-test, a final questionnaire will be prepared and made accessible electronically on the secure website. All potential participants will be sent an information sheet about the project as well as a consent form by both mail and email. After creating their personalised electronic account by entering their username and choosing their password, participants will be guided through the process of the electronic Delphi questionnaire. Participants will be asked to rate the applicability and the importance of each proposed items on a seven-point Likert scale.

Results from the first round will be compiled and a mean score of applicability and importance for each item will be calculated. Then, participants will be invited to participate in a second round rating process by email and postcard, through the secure website [53,54]. Experts will again be asked to rate the degree of applicability and importance of each identified factors, having the mean score for each item from the previous round. Participants will also be able to add free text comments. Email and postcard reminders will be sent to non-responding participants after in each round. A third round survey, based on the responses of the second round, might be necessary if a consensus is not reached for at least 70% of the items [55]. Finally, the consensual rating will be sent a last time to the experts for a final validation.

#### Analysis of ratings

Aggregate ratings will be calculated and feedback comments will be content analyzed for each rounds of the survey. Also, to ensure equal weighting for each experts group in the overall rating, a weighted median will be calculated. A satisfactory degree of consensus will be obtained if less than 30% of the ratings are in the lower range (ratings one or two) and less than 30% of the ratings are in the upper range (ratings six or seven) [53,54,56].

#### Ethical considerations

All data collected for the document analysis in this study will be obtained from publicly available sources. Participants to the Delphi study will be given specific consent forms presenting research objectives and information about research implications. They will be informed that their participation to the study is entirely voluntary and that they implicitly consent to participate when creating their electronic account. Ethics approval for the study protocol has been received from the Research Ethics Board of the Centre Hospitalier Universitaire de Québec (approved 23 January 2009; ethics number 5-08-12-06).

#### Knowledge translation plan

This synthesis aims at producing usable knowledge that could support decision makers responsible for the implementation of interoperable EHR in Canada. As such, the first group that will be targeted by the knowledge translation activities will be decision makers who collaborate to the project. Researchers of the team will present the key messages at meetings of the Infoway Innovation and Adoption Committee. Also, collaborators of the team representing groups of EHR end-users (physicians, nurses, pharmacists, medical archivists, patients, and citizens) will be invited to the scientific lunch presentations at the Quebec City University Hospital research centre that we will organize after the completion of each phase of the research. Key messages will also be sent to experts who have participated in the Delphi study for diffusion to their communities. Documents will be written in a non-technical language and in a '1-3-25' format which is considered as a successful way to reach busy policy makers [57].

Entities responsible for the implementation of EHR, such as provincial health ministries and regional health authorities will be our primary external audiences. We will present our final result at national conferences on EHR that gathers representatives from all provinces, such as the Transforming Government: better OUTCOMES for Citizens Conference. Also, key messages will be sent to journals of healthcare professional associations and we will solicit interviews with their journalists. We will also capitalize on provincial conferences gathering healthcare decision makers.

The results of this synthesis will also be published in scientific journals that offer online access, as well as in relevant professional journals. Results will be presented at national and international conferences with a focus on the EHR, such as the American Medical Informatics Association conference.

## Discussion

This project directly aligns with one of the Canadian Institutes of Health Research strategic priority research areas that emerged from 'Listening for Direction III: Health Information' [58]. There is an urgent need to establish an information infrastructure to support reform efforts of Canada's healthcare system [58], and initiatives to build this health information infrastructure include Infoway's efforts, together with provincial initiatives, to create and implement EHR [59]. However, in spite of these initiatives, significant gaps remain in understanding the challenges related to developing, implementing, and maintaining health information systems. Understanding these challenges is critical to support future investments, encouraging clinicians to adopt EHR solutions, ensuring public confidence in the system, and using the information to inform policy and planning [58].

This knowledge synthesis will lead to a proposed set of strategies for effective implementation of EHR in Canada by identifying its barriers and facilitators. This knowledge is also central to Infoway's business plan because the benefits that are expected with EHR are dependent upon its effective implementation and utilization by many groups of users [3]. Evidence is needed to prepare this major shift in our healthcare system and to oversee the factors that could affect its adoption and integration by all its potential users. This study is being conducted in close collaboration with decision makers from Infoway which promotes knowledge-sharing and its application to support decisions in real life context. Therefore, our results are likely to be used in order to inform decision makers about strategies for an optimal implementation of EHR in the Canada health care system.

This study will be the first, to the best of our knowledge, to systematically summarize the barriers and facilitators to EHR adoption perceived by different groups and to consider the local contexts in order to ensure the applicability of this knowledge to the particular realities of various Canadian jurisdictions. It aims to produce knowledge that is relevant, timely, and useful for decision makers who are directly responsible for the optimal integration of the EHR in all Canadian jurisdictions. Finally, the systematic approach undertaken and the rigorous methods that we will follow are likely to be transferable to other settings in order to explore context-relevant factors of EHR adoption across jurisdictions.

## Competing interests

The authors declare that they have no competing interests.

## Authors' contributions

All authors collectively drafted the research protocol and approved the final manuscript. MPG is its guarantor.
